# Rural–urban disparities and factors associated with delayed care-seeking and testing for malaria before medication use by mothers of under-five children, Igabi LGA, Kaduna Nigeria

**DOI:** 10.1186/s12936-020-03371-w

**Published:** 2020-08-18

**Authors:** Obafemi J. Babalola, Olufemi Ajumobi, IkeOluwapo O. Ajayi

**Affiliations:** 1 Liberia Field Epidemiology, Laboratory Training Programme, National Public Health Institute, Congo Town, Montserrado, Liberia; 2 Nigeria Field Epidemiology and Laboratory Training Programme (NFELTP), Asokoro district, Abuja, Nigeria; 3 Department of Epidemiology and Medical Statistics, College of Medicine, University of Ibadan, Ibadan, Oyo State Nigeria; 4 National Malaria Programme, Federal Ministry of Health, Abuja, Nigeria; 5 School of Community Health Sciences, University of Nevada Reno, Reno, NV USA

**Keywords:** Malaria, Mothers, Fever, Treatment-seeking, Under-fives, Knowledge, Nigeria

## Abstract

**Background:**

Fever in under-five children (U5) is the commonest presenting complaint in general practice and mothers’ recognition is an entry point for fever treatment, including malaria. This study describes rural–urban disparity in fever prevalence in U5, mothers’ malaria knowledge, care-seeking, testing for malaria before anti-malarial medication and the associated factors.

**Methods:**

A cross-sectional survey was conducted among 630 mother–child pairs [rural (300) and urban (330)] selected randomly using a multi-stage sampling from 63 villages in Igabi LGA, Kaduna State, Nigeria. Trained female data collectors administered a pre-tested structured questionnaire to collect information on mother–child demographic profiles, malaria knowledge, fever episodes in birth order last child in two weeks prior to survey, blood testing before anti-malarial use, and delayed care-seeking defined as care sought for fever > 48 h of onset. Malaria knowledge was categorized into good, average, and poor if the final scores were ≥ 75th, 50th–74th, and < 50th percentiles, respectively. Frequency, proportions, and odds ratio were calculated. Statistically significant was set at p-value < 0.05.

**Results:**

The median age (interquartile range) of rural mothers was 30 (IQR, 10) years compared to 27 (IQR, 6) years in urban. Of the 70.0% (441/629) U5 children with fever, 58.5% (258/441) were in rural settlements. A third of the mothers whose child had fever sought care. Mothers in rural settlements were 2.8 (adjusted OR: 2.8, CI 1.8–4.2, p < 0.01) times more likely to delay care-seeking for fever. Other significant factors were poor or no knowledge of malaria transmission, poor perception of malaria as a major health problem, and household size > 5. Also, mothers who had no formal education were four times more likely to receive anti-malarial medications without testing for malaria compared to their educated counterpart (adjusted OR: 4.0, 95% CI 1.6–9.9, p < 0.000).

**Conclusions:**

Rural–urban disparities existed between fever prevalence in U5 children, care-seeking practices by their mothers, and factors associated with delayed care-seeking and testing the fever for malaria before anti-malarial medication. Fever treatment for high impact malaria elimination in Nigeria needs a context-specific intervention rather than ‘one-size-fits-all’ approach.

## Background

Globally, 228 million malaria cases and 405,000 deaths were reported in 2018; India and 18 African countries accounted for 85% of these cases [1]. Children were more vulnerable with 67% of deaths in those under the age of five years [1]. In 2018, Nigeria was the first among the 11 countries with high malaria burden globally, accounting for 25% of cases and 24% of deaths globally [1]. Between 2015 to 2018, Nigeria reported a one-fifth reduction (20%) in the proportion of under-five children (U5) that were positive for malaria using the malaria rapid diagnostic test (RDT) from 45% in 2015 [2] to 36% in 2018 [3]. However, 40% of the population at risk are protected from mosquitoes bite by sleeping under long-lasting insecticidal nets (LLINs) [1].

Recently, data have shown a global reversal in the gains of malaria control [4]. Malaria accounts for a significant burden of febrile illnesses in endemic areas [5] and to achieve the vision of malaria elimination, a country-led ‘High burden to High impact’ approach was recommended by the World Health Organization (WHO) in 2018. The approach was aimed at redirecting the static global malaria control response with a focus on high malaria-burdened countries for impact. The pillar two of this approach emphasized the use of strategic information rather than one-size-fits-all approach to drive this impact. This encouraged sub-national, evidence–based, strategic information and interventions to drive impact for improve malaria elimination [4]. This underscores localized effort with focus on high-burden settings where intensified malaria interventions are targeted at a drastic reduction in malaria cases and deaths.

In Nigeria, as in other tropical countries, fever serves as a useful marker for recognition of infections in U5 children, including malaria [6, 7]. Fever is a primary complaint for a third of all paediatric consultations in general practice [6, 8], and an entry point for parents or mothers to seek medical attention for U5 children, including malaria diagnosis and case management [7, 9, 10]. The prevalence of fever in U5 children in Kaduna State, Nigeria in 2018 was 26% [3]. Although fever is not a pathognomonic sign of malaria, it usually trigger mothers, being the most-at-home in Nigeria to take further action in seeking appropriate care, which may eventually be diagnosed as malaria [1].

The health-seeking behaviour for febrile illness by mothers of U5 children is poor and not commensurate with high burden of fever and malaria in Nigeria, a country with highly favourable climatic conditions for malaria transmission [1, 2, 4, 11–13]. The Nigeria Demographic Health Survey 2018 revealed that 29% of mothers with no education had the highest proportion of children with a fever in the previous 2 weeks [3] and six out of ten mothers sought treatment from private sectors, such as patent medicine vendors and pharmacists. Additionally, like other high malaria-endemic countries, home self-medication is a common response of Nigeria mothers to fever [6, 11, 13]_._

Several factors that differs from one locality to another and within sub-populations may contribute to decision on how, when, and where mothers of U5 children seek treatment and test fever for malaria before using anti-malarial drugs. Some of these factors are mothers’ knowledge of the causes of fever [10], perception of fever as indicator of illness severity, availability and accessibility to skill community health workers and health facilities [14]. Other factors are short duration of fever and rapid recovery [1], the season (rainy season) of the fever which coincides with high prevalence of malaria, rural location, availability of malaria commodities in the health facilities, mother’s level of education [10, 15], lack of money for transportation and other expenses to treat the fever, the age and gender of the child [16], perception of the family decision-maker, mother’s employment status [17], household size > 4 members [12], and hospital waiting time [13, 18–21].

In 2018 National Demographic Health Survey, despite several measures put in place to reduce the prevalence of fever, fever was reported in a quarter of U5 children in Kaduna Nigeria in the previous two weeks preceding the survey and fewer number had their blood tested for malaria before anti-malarial medication. Therefore, to develop a context-specific approach to fever treatment and drive high impact malaria elimination process in Igabi LGA Kaduna Nigeria, this study will provide information for programmatic intervention development. It aims to describe rural–urban disparity in fever prevalence, mothers’ malaria knowledge, and associated factors with delayed care-seeking for fever in U5 and testing fever for malaria before anti-malarial drugs.

## Methods

### Study area

Igabi Local Government Area (LGA) with headquarters at Turunku is one of the 23 LGAs in Kaduna State, North-west geopolitical zone, Nigeria. It has a population of 557,624 and is sub-divided into five urban districts: Afaka, Birnin-yero, Kwarau, Rigachikun, and Rigasa, and seven rural districts: Fanshanu, Gwaraji, Igabi, Kerawa, Sabo-Birnin, Turunku, and Zangon Aya. The LGA is located 650 m above sea level, between latitude 10° 47ʹ 0ʺ N and longitude 7° 46ʹ 0ʺ E in the tropical Sahel to Sudan Savannah, with annual rainfall varying from 1000 to 1500 mm and highest precipitation of 72% in August. The rainy season and period of high malaria transmission is usually from June to October. The annual mean temperature is 34 °C. This can rise to 41 °C at the peak of the dry season in April and drop as low as 12 °C in January during severe *harmattan*. The prevalence of malaria cases diagnosed microscopically among U5 children in Kaduna State is 36.7% [2]. This study employed a cross-sectional design.

### Study design and population

This was a part of a larger study that has been published, and addressed the characteristics of women of child bearing age (WCBA) associated with long-lasting insecticidal nets (LLINs) ownership and utilization [22]. Moreover, the current analysis focused on rural–urban disparity in fever prevalence, mothers’ malaria knowledge, and associated factors with delayed care-seeking for fever in U5 children and fever testing for malaria before anti-malarial medication. Mothers and their U5 children with at least 12 months’ residence in the selected households in the community were included. Anyone with cognitive deficits, or any chronic and debilitating illness that hinders effective participation in an interview was excluded.

### Sample size determination

Using the formula, n = z^2^pq/d^2^ at 95% confidence interval (1.96), the percentage for children with fever for whom advice or treatment was sought in North-Western Nigeria is 63.6% [23] with a confidence limit of ± 5%, and a non-response rate of 10%. To adjust the required sample size for cluster sampling design, a correction factor of 1.5 design effect was used as fewer heterogenous communities in this district was assumed. The calculated minimum sample size was 615. However, a minimum sample size of 630 was used to accommodate the sample size for the WCBA study [22].

### Sampling technique

Participants were selected using multi-stage sampling technique. The sampling technique has been described elsewhere [22]. In Stage 1, Igabi LGA was stratified into rural and urban wards and ten wards were randomly selected. These included six rural wards: Fanshanu, Gwaraji, Igabi, Sabo-Birnin, Turunku, and Zangon Aya, and four urban wards: Afaka, Birnin-yero, Rigachikun, and Rigasa. For the selection of a cluster or village/settlement in stage 2, household enumeration data generated by WHO/UNICEF during micro-planning for the mass LLIN campaign in these settlements was used as the sampling frame. With a sample size of 630, to improve the validity and precision of estimates and considering a wide community representation (population variance), using a probability proportional to size, 63 villages or clusters (i.e., 30 rural and 33 urban) were randomly selected with a cluster size of 10. Using the list of the households in each village/settlement generated during micro-planning for mass LLIN distribution, ten households were systematically selected from each village/settlement at stage 3. One mother from each was interviewed in a household. In a selected household with multiple eligible mothers of U5 children, one was randomly selected by the ballot and recruited into the study. The last childbirth order of the eligible mothers was recruited into the study and if the last child was a set of twins, one of the twins was randomly selected by balloting.

### Data collection

Data were collected by ten trained female community health extension workers and nurses who speak English and Hausa languages fluently and reside within the district from September to October 2015 as described elsewhere [22]. They were supervised by five undergraduate medical students. The data collection tool was a pretested, structured questionnaire adapted from the Malaria Indicator Survey [24, 25] and other literature [26, 27]. This was used to collect information on demographic profile, mothers’ knowledge of malaria, history of fever onset in U5 children and mothers’ fever treatment-seeking behaviour within 48 h of fever onset. The malaria knowledge items were questions from the literature, malaria indicator surveys and demographic health surveys. The questionnaire’s face and content validity, item’s accuracy, relevance, and clarity has been described elsewhere [22]. The knowledge of malaria was assessed using five thematic areas including cause and mode of transmission, mosquito feeding time, symptoms, diagnosis, and prevention. The knowledge of malaria questions was open ended with no response provided and if provided, they are not read to the participants. Any participants who correctly responded that mosquito bites cause malaria transmission to humans, mosquito feeding time was night-time, symptoms are fever and any other symptoms, and prevention by using either LLINs, indoor residual spray (IRS) or mosquito repellent coils were given ‘1’ and incorrect responses scored ‘0’. The individual score was calculated by finding a percentage of total score obtained from maximum allowable score of 6 and knowledge of malaria was categorized using percentile scores. Good knowledge of malaria, if final score falls at 75th percentile or more, score between 50 and 74th percentile as average knowledge and poor knowledge if score was < 50th percentile.

### Study variables

The primary outcome or dependent variable for this study was dichotomized to know if mothers sought care for fever or not within 48 h of onset in U5 children. The secondary outcome variable was also dichotomized to know whether the U5 child receive a recommended care for malaria fever or not. The recommended care for malaria fever was defined as blood testing for malaria parasites before artemisinin-based combination therapy (ACT) medication and care for fever received from skilled community health care workers within 48 h of fever onset. Independent and explanatory variables were U5 children and respondents’ characteristics and mothers’ knowledge of malaria. Mothers assumed presence of fever by tactile palpation of children’s skin and felt the hotness beyond normal. However, any fever episode in U5 children two weeks prior to the survey was taken as history of fever from which questions were asked from the mothers to know if care was sought within 48 h of onset or not. Delayed care-seeking for fever was the inability to seek care for fever within 48 h of fever onset [28].

### Data processing and analysis

Data were entered and analysed using Epi-Info version 7 statistical software. Frequency and proportions were calculated for socio-demographic characteristics, knowledge of malaria, presence of fever in U5 children in previous two weeks, and mothers seeking care or treatment for fever within 48 h of onset, and obtaining a recommended care of fever testing before anti-malarial medication. Also, median (range) for continuous descriptive variables was calculated as well as frequency and proportions for categorical variables. Chi-squared test was used to test for association between dependent outcome and independent categorical variables; results were presented in odd ratios at 95% confidence interval (CI). The objective upward loading method was used for multivariable logistic regression modelling and associated factors with p value ≤ 0.2 and adjusted Odd ratio were selected in the model to identify the predictors of delayed care-seeking and receiving a recommended care for malaria fever. Results of all statistical analyses were considered significant at p-value of < 0.05.

## Results

### Mothers and U5 children’s characteristics

Of the sampled 630 mothers-child pairs, 629 (99.8%) completed the interview and were included in the analysis, 258 (41.1%) were employed, and 300 (47.7%) were living in rural communities. Rural mothers were older, the median age (interquartile range, IQR) was 30 years (IQR, 10) to 27 years (IQR, 6) of their urban counterpart. Also, more rural mothers had no education with 61.7% (185/360) versus 21.3% (70/329) in urban mothers, but 61.7.% (203/329) of the later had secondary level education compared to 38.3% (115/300) rural mothers. Rural mothers were significantly different from the urban mothers in age (p = 0.001), educational level (p = 0.001) and occupations (p = 0.001). The median age (IQR) of the U5 children was 12 months (IQR, 12). Urban children were younger, with median age (IQR) 12 months (IQR 12), while the median age of rural children was 24 months (IQR 12) months. Male children were 56.4% (355/629).

### Knowledge of malaria

Overall, 139 (22.1%) respondents have good malaria knowledge and no significant difference (p = 0.05) in the knowledge of malaria between the rural and urban women. However, on each malaria knowledge items, 553 (87.9%) mothers knew that malaria is transmitted to humans through the bites of infected mosquitoes, 432 (68.7%) knew that malaria causing mosquitoes commonly feed on humans at night-time, 527 (83.8%) knew malaria symptoms, and 381 (60.6%) knew that malaria RDT kit and/or microscopy are useful in detecting if malaria-causing organisms (parasites) is the cause of a fever, and malaria prevention with the use of LLINs, IRS and mosquito repellent coils were mentioned by 342 (54.4%) mothers. There was a significant difference between the rural and urban mothers on the malaria knowledge items as shown in Table 1. More urban women, 276 (83.9%) versus 156 (52.0%) rural women knew that malaria mosquitoes feeding time was night-time. However, less urban mothers, 128 (38.9%) compared to 158 (52.7%) rural mothers knew that testing of blood with malaria RDT to detect malaria parasite as the cause of fever in U5 children is necessary before anti-malarial medication. Associated with mothers’ poor knowledge of malaria was having no formal education (OR: 1.8, 95% CI 1.3–2.6, p < 0.001) and the perception of malaria as not a major health problem in the community (OR: 2.5, 95% CI 1.5–4.4, p = 0.001).

**Table 1:** Knowledge of malaria among respondents by location of residence (rural/urban), Igabi LGA, Kaduna Nigeria, (N = 629)

Knowledge characteristics	Rural (n = 300)	Urban (n = 329)	Chi square	p-value
Overall knowledge of malaria				
Good	54 (38.8)	85 (61.2)	5.9	0.05
Average	102 (48.8)	107 (51.2)		
Poor	144 (51.3)	137 (48.7)		
Malaria transmission route to humans				
Bite of malaria infected mosquito	261 (87.0)	292 (88.8)	10.9	< 0.01
Do not know	30 (10.0)	15 (4.5)		
Through contaminated water/foods	9 (3.0)	22 (6.7)		
Malaria mosquito feeding time				
Both day and night-time	73 (24.3)	44 (13.4)	89.2	< 0.01
Day time	11 (3.7)	4 (1.2)		
Do not know	60 (20.0)	5 (1.5)		
Night-time	156 (52.0)	276 (83.9)		
Malaria diagnosis				
Blood microscopy	47 (15.7)	48 (14.6)	37.8	< 0.01
Do not know	37 (12.3)	19 (5.8)		
Symptomatically	58 (19.3)	134 (40.7)		
Testing blood with malaria RDT	158 (52.7)	128 (38.9)		

			OR (95% C.I)	p-value
Malaria symptoms (yes/no) reference, no				
Fever	269 (89.7)	309 (93.9)	0.6 (0.3–1.0)	0.07
Headache	217 (72.3)	262 (79.6)	0.7 (0.5–0.9)	0.04
Vomiting	164 (54.7)	188 (57.1)	0.9 (0.7–1.2)	0.59
Shivering	162 (54.0)	119 (36.2)	2.1 (1.5–2.8)	< 0.01
Diarrhoea	50 (16.7)	38 (11.5)	1.5 (0.9–2.4)	0.08
Dizziness	44 (14.7)	115 (35.0)	0.3 (0.2–0.5)	< 0.01
Nausea	40 (13.3)	86 (26.1)	0.4 (0.3–0.7)	< 0.01
Loss of appetite	28 (9.3)	93 (29.2)	0.2 (0.1–0.4)	< 0.01
Malaria prevention (yes/no) reference, no				
Clearing of grasses/breeding sites	197 (65.7)	228 (69.3)	0.8 (0.6–1.2)	0.37
Use long lasting insecticidal nets	108 (36.0)	144 (43.8)	0.7 (0.5–1.0)	0.05
Use mosquito coil or repellent	51 (17.0)	90 (27.4)	0.5 (0.4–0.8)	< 0.01
Wearing long sleeve shirts	9 (3.0)	10 (3.04)	1 (0.4–2.5)	1.00
Indoor residual spray	1 (0.3)	66 (20.1)	0.01 (0.0–0.1)	< 0.01

### Fever occurrence in U5 children and mothers’ care-seeking practice

Of the 441 (70.0%) U5 children with fever episode in previous two weeks before survey, mothers sought care within 48 h of onset for 155 (35.2%). In Table 2, fever in previous two weeks in U5 children was more commonly reported by rural mothers 258/300 (86.0%) compared to 183/329 (55.6%) urban mothers. In contrast, fewer rural mothers 63/258 (24.4%) compared to 92/183 (50.3%) urban mothers sought care for fever within 48 h of onset. Of the 155 mothers who sought care for fever within 48 h of onset, 52 (33.5%) did home self-medication, 28 (18.1%) sought care at general hospital, 27 (17.4%) went to the pharmacy or patent medicine vendor’s shop, 17 (11.0%) visited private hospitals and 5 (3.2%) sought advice from religious leaders and traditional/herbal treatment home. Among those that sought care, 70 (45.2%) were given ACT to treat fever, 48 (31.0%) U5 children had their blood tested for malaria parasite, and 94 (60.6%) was hospitalized (Table 2).

**Table 2:** Fever in U5 children and mothers’ care-seeking practice for fever, Igabi LGA, Kaduna Nigeria

Characteristics	Rural (n = 300)	Urban (n = 329)	OR (95% C.I)	p value
Child had fever (n = 629)				
Yes	258 (86.0)	183 (55.6)	4.9 (3.3–7.3)	< 0.01
No	42 (14.0)	146 (44.4)	Ref	
Mother sought care < 48 h (n = 441)				
No	195 (75.6)	91 (49.7)	3.1 (2.1–4.7)	< 0.01
Yes	63 (24.4)	92 (50.3)	Ref	
Had recommended care (n = 155)				
Yes	28 (44.4)	20 (21.7)	2.9 (1.4–5.8)	0.00
No	35 (55.6)	72 (78.3)	Ref	
Child blood tested for malaria (n = 155)				
Yes	28 (44.4)	20 (21.7)	2.9 (1.4–5.8)	0.01
No	35 (55.6)	72 (78.3)	Ref	
Child given drugs (n = 155)				
Yes	57 (90.5)	87 (94.6)	0.5 (0.2–1.9)	0.51
No	6 (9.5)	5 (5.4)	Ref	
Child hospitalised (n = 155)				
Yes	36 (57.1)	58 (63.0)	0.8 (0.4–1.5)	0.57
No	27 (42.9)	34 (37.0)	Ref	

			Chi Square	p value
Where care was sought for fever (n = 441)				
Health facility	28 (10.8)	38 (20.8)	36.5	< 0.01
Religious leaders, herbal	2 (0.8)	8 (4.4)		
Pharmacy store/patent med. vendors	15 (5.8)	12 (6.5)		
Self-treatment at home	18 (7.0)	34 (18.6)		
Not seek help	195 (75.6)	91 (49.7)		
Type of malaria drugs given (n = 155)				
Artemisinin-based combination therapy	36 (57.1)	34 (37.0)	7.1	0.03
Sulfadoxine/pyrimethamine	22 (34.9)	52 (56.5)		
No anti-malarial drugs	5 (8.0)	6 (6.5)		

With reference to urban communities (Table 3), rural children were five times (OR: 4.9, 95% CI 3.3–7.3, p < 0.001) more likely to have fever episodes in previous two weeks, rural mothers were thrice more likely (OR: 3.1, 95% CI 2.1–4.7, p < 0.001) to delay (> 48 h from fever onset) care-seeking for fever, and rural mothers were thrice more likely (OR: 2.9, 95% CI 1.4–5.8, p = 0.001) to have the blood of their children tested for malaria before taking anti-malarial medications.

**Table 3:** Factors associated with delayed care-seeking for fever by mothers of U5 children, Igabi LGA, Kaduna Nigeria, (N = 441)

Characteristics	Delayed care-seeking		OR (95% C.I)	p value
	Yes	No		
Mother’s age < 30 years				
No	164 (69.2)	73 (30.8)	1.5 (1.0–2.2)	0.05
Yes	122 (59.8)	82 (40.2)	Ref	
Type of community				
Rural	195 (75.6)	63 (24.4)	3.1 (2.1–4.7)	0.00
Urban	91 (49.7)	92 (50.3)	Ref	
Mother had formal education				
No	150 (72.1)	58 (27.9)	1.8 (1.2–2.7)	0.00
Yes	136 (58.4)	97 (41.6)	Ref	
Religion				
Islam	275 (64.4)	152 (35.6)	0.5 (0.1–1.8)	0.40
Christianity	11 (78.6_	3 (21.4)	Ref	
Household size < 5				
Yes	127 (57.2)	95 (42.8)	0.5 (0.3–0.7)	0.00
No	159 (72.6)	60 (27.4)	Ref	
Malaria, a major health problem				
No	40 (81.6)	9 (18.4)	2.6 (1.2–5.6)	0.01
Yes	246 (62.8)	146 (37.2)	Ref	
Mother knows mode of malaria transmission				
No	27 (57.4)	20 (42.6)	0.7 (0.4–1.3)	0.30
Yes	259 (65.7)	135 (34.3)	Ref	
Mother knows mosquitoes feeding time				
No	111 (72.6)	42 (27.4	1.7 (1.1–2.6)	0.02
Yes	175 (60.8)	113 (39.2)	Ref	
Mother knows malaria symptoms				
No	54 (60.7)	35 (39.3)	0.8 (0.5–1.3)	0.40
Yes	232 (65.9)	120 (34.1)	Ref	
Mother knows how to detect if fever is caused by malaria				
No	97 (61.8)	60 (38.2)	0.8 (0.5–1.2)	0.40
Yes	189 (66.5)	95 (33.5)	Ref	
Mother knows malaria prevention measures				
No	142 (61.2)	90 (38.8)	0.7 (0.5–1.1)	0.10
Yes	144 (68.9)	65 (31.1)	Ref	
Poor malaria knowledge				
Yes	139 (69.9)	60 (30.2)	1.5 (1.0–2.2)	0.06
No	147 (60.7)	95 (39.3)	Ref	
Total	286 (64.8)	155(35.2)		

### Factors associated with delayed care-seeking for fever in U5 children and obtaining a recommended care of fever for malaria

Factors associated with delayed care-seeking for fever in U5 children was shown in Table 3. Delayed care-seeking for fever in U5 children was positively associated with no formal education for mothers (OR: 1.8, CI 1.2–2.7, p = 0.003), perception of malaria as not a major health problem in the community (OR: 2.6, CI 1.2–5.6, p = 0.01), and poor knowledge of mosquitoes’ feeding time (OR: 1.7, CI 1.1–2.6, p = 0.02). Also, delayed care-seeking care for fever in U5 was negatively associated with household size of < 5 persons. Mothers in household size of < 5 persons were 50% less likely (OR: 0.5, 95% CI 0.3–0.7, p < 0.00) to delay care-seeking for fever. In Table 4, receiving a recommended care by testing blood for malaria parasite before anti-malarial medications was associated with rural mothers. Rural mothers were thrice likely (OR: 2.9, 95% CI 1.4–5.8, p = 0.00) to test blood for malaria parasite before anti-malarial medications compared to their urban counterparts. However, mothers with no formal education were 70% (OR: 0.3, 95% CI: 0.1–0.7, p = 0.01) less likely to test U5 children’s blood for malaria parasite before anti-malarial medications compared to mothers with formal education.

**Table 4:** Factors associated with caregivers of U5 children receiving a recommended care of testing fever for malaria before using anti-malarial, Igabi LGA, Kaduna Nigeria. (N = 155)

Characteristics	Test blood for malaria parasite before taking anti-malarial drugs			p value
	Yes	No	OR (95% C.I)	
Mother’s age < 30 years				
No	22 (30.1)	51 (69.9)	0.9 (0.5–1.8)	0.97
Yes	26 (31.7)	56 (68.3)		
Type of community				
Rural	28 (44.4)	35 (55.6)	2.9 (1.4–5.8)	0.00
Urban	20 (21.7)	72 (78.3)		
Mother’s had formal education				
No	10 (17.2)	48 (82.8)	0.3 (0.1–0.7)	0.01
Yes	38 (39.2)	59 (60.8)		
Religion				
Islam	47 (30.9)	105 (69.1)	0.9 (0.1–10)	1.00
Christianity	1 (33.3)	2 (66.7)		
Household size < 5				
Yes	24 (25.3)	71 (74.7)	0.5 (0.3–1.0)	0.08
No	24 (40.0)	36 (60.0)		
Malaria, a major health problem				
No	2 (22.2)	7 (77.8)	0.6 (0.1–3.1)	0.83
Yes	46 (31.5)	100 (68.5)		
Mothers knows mode of malaria transmission				
No	2 (10.0)	18 (90.0)	0.2 (0.1–1.0)	0.06
Yes	46 (34.1)	89 (65.9)		
Mothers knows mosquitoes feeding time				
No	15 (35.7)	27 (64.3)	1.3 (0.6–2.9)	0.55
Yes	33 (29.2)	80 (70.8)		
Mothers knows malaria symptoms				
No	11 (31.4)	24 (68.6)	1.0 (0.5–2.3)	1.00
Yes	37 (30.8)	83 (69.2)		
Mothers knows how to detect if fever is caused by malaria				
No	16 (26.7)	44 (73.3)	0.7 (0.4–1.4)	0.46
Yes	32 (33.7)	63 (66.3)		
Mothers knows malaria prevention measures				
No	21 (32.3)	44 (67.7)	1.1 (0.6–2.2)	0.89
Yes	27 (30.0)	63 (70.0)		
Poor malaria knowledge				
Yes	16 (26.7)	44 (73.3)	0.7 (0.4–1.5)	0.46
No	32 (33.7)	63 (66.3)		
Total	48 (31.0)	107 (69.0)		

Table 5 shows the predictors of delayed care-seeking and blood testing for malaria before anti-malarial medications. Although, rural mothers were thrice more likely to delay care-seeking for fever in U5 children (adjusted OR: 2.8, CI 1.8–4.2, p < 0.01) than urban mothers, but were 80% less likely to use anti-malarial medications without blood testing for malaria parasite in U5 children with fever (adjusted OR: 0.2, 95% CI 0.1–0.4, p < 0.00). Mothers with no formal education were four times (adjusted OR: 4.0, 95% CI 1.6–9.9, p < 0.000) likely to received unrecommended care for malaria fever, i.e., use anti-malarial medications without blood testing for malaria parasite in U5 children with fever, compared to those with formal education. On the other hand, mothers in households size of < 5 persons were 60% less likely to delay care-seeking compared to mothers in large household size of 5 or more persons (adjusted OR: 0.4, 95% CI 0.3–0.7, p < 0.000).

**Table 5:** Predictors of delayed care-seeking for fever and receiving a recommended care of testing blood for malaria parasite before using anti-malarial, Igabi LGA, Kaduna Nigeria

Term	Delayed care-seeking		
	Adjusted Odds Ratio (95% C.I)	Coefficient	p-value
Mother's age ≥ 30 years	1.5 (1.0–2.4)	0.4122	0.072
Rural settlement	2.8 (1.8–4.2)	1.0181	0.000
Household size < 5 people	0.4 (0.3–0.7)	− 0.8269	0.000
Poor malaria knowledge	1.4 (0.9–2.2)	0.3656	0.090

Term	Unrecommended care of malaria fever		
	Adjusted Odds Ratio (95% C.I)	Coefficient	p-value
Not knowing route of malaria transmission to human	5.4 (1.1–26.7)	1.6925	0.040
Rural settlement	0.2 (0.1–0.4)	− 1.6854	0.000
No formal education	4.0 (1.6–9.9)	1.3831	0.001

## Discussion

This study describes rural–urban disparities in the prevalence of fever in U5 children, mothers’ knowledge of malaria, factors associated with delayed care-seeking for fever and receiving a recommended care for malaria fever in U5 children at Igabi LGA, Kaduna State, Nigeria. The reported disparities were in the age of U5 children and their mothers, the urban mothers were more educated, currently employed, and more knowledgeable on malaria. The study also highlighted higher prevalence of fever among rural U5 children, delayed care-seeking for fever by their mothers who were likely to receive a recommended care for malaria fever, but unlike mothers with no formal education who were likely to receive un-recommended care for malaria fever.

Although, living in rural settlement was found to associate with low level of education and this may influence the poor knowledge of malaria [1] reported among the rural women in this study. In line with previous literature, urban mothers were more exposed to formal education and earlier, they have access to newspapers, television, internet and social media for health information [3]. These various means of knowledge acquisition platforms would have influenced urban mothers’ knowledge of fever and malaria and improve their perception to know that malaria is a major health problem in their community. This study shows that mothers’ perception of malaria as not a major health problem was significantly associated with poor malaria knowledge. Mothers’ knowledge of malaria and recognition of fever in U5 children is vital to activate appropriate care-seeking behaviour for treatment of fever [15], but the study found no significant differences in the overall knowledge of malaria between rural and urban women. Although higher proportion of urban women had good or average knowledge of malaria but having no formal education was significantly associated with poor malaria knowledge. The poor malaria knowledge among rural women may account for the higher prevalence of fever in U5 children and may influence mothers care-seeking habits. In contrast, good knowledge of malaria among urban mothers does not translate to receiving a recommended care for malaria fever in U5 children. Generally, mothers in Nigeria accept care presented by care providers without demanding for a recommended care which favours a presumptive treatment.

In Nigeria, fever episode is usually common in rural communities as corroborated in this study [2] with three out of four U5 children having fever episodes in previous two weeks. Fever is a useful marker for mother’s recognition of infections in U5 children, including malaria [6, 7]. Therefore, it serves as entry point for mothers to seek care, including malaria diagnosis and case management [7, 9, 10]. However, educational level of mothers may influence decision to seek care for fever in U5 children within 48 h of onset. Fever episode reported in this study was higher than a quarter reported in the 2018 DHS and 52.1% reported in 2015 Malaria Indicator Survey (MIS) for North West Geopolitical Zone, Nigeria [2], the survey that was done at the same time as this study.

Mothers living in urban communities sought care for fever in U5 children earlier than rural mothers [3]. This study shows that mothers who sought care for fever episode within 48 h of onset in U5 children was similar to 31.4% reported in 2015 MIS, but this was lower than a study in Northern Nigeria that reported 61.5% [19], and 73% reported for Kaduna State in DHS 2018 [3]. But this was higher than 28.6% reported in Ethiopia [10]. Approximately, four out of ten mothers sought care from a skilled health worker in hospital or primary health centre, similar to other studies from Northern Nigeria [17, 19], but higher than 28% in 2018 DHS [3]. Also, this study like others reported similar proportions of mothers who sought care at pharmacy shops or patent medicine vendors [12, 15, 16, 18, 29]. Although some mothers resorted to self-medication at home and this proportion was lower compared to a study from south-eastern Nigeria [18] and Myanmar [16]. Apart from the place of residence i.e., urban, good knowledge of malaria transmission and prevention has been found to be associated with care-seeking for fever treatment from a skilled health workers [21], self-medication at home for fever in U5 children has also been associated with mothers’ level of education [1]. Asides from levels of education, the distance to health facility, access to community health worker, transportation cost, presumptive self-treatment of fever, household poverty, cultural norms or household mechanism for authorization on care-seeking, large household size with five or more persons, perception of malaria as not a major health problem were factors associated with mothers delaying seeking advice or care for treatment of fever episode in U5 children within 48 h of onset [7, 10, 12–14, 19, 28].

In rural settlements, mothers are likely not to seek care for fever due to their poor exposure to formal education and health information [8]. Although, this study shows that rural mothers were likely to delay care-seeking they were more likely to receive recommended care of fever by testing blood for malaria before using anti-malarial drugs. This could be attributed to free malaria diagnosis and treatment strategy implemented by the Government of Kaduna State with widespread distribution of malaria RDT and artemisinin-based combination medicines in community health centres and patronage of the community health centres by rural women. On the other hand, urban women have more access to private health facilities, pharmacy shops, and patent medicine vendors that concentrate and render services in urban communities. These sets of care providers commonly practice presumptive malaria treatment and prescribed un-recommended drugs (no longer recommended for the treatment of malaria fever). Other reasons for not testing blood for malaria parasite before anti-malarial medication in U5 children could be attributed to self-treatment of fever at home by urban women, no formal education, care received from health workers that did not know the current treatment strategy for malaria in Igabi LGA, Kaduna State, and unavailability of malaria diagnostic kits, drugs and other commodities in the health facility or pharmacy shops in the community.

This study shows that different factors are responsible for mothers delaying care-seeking for fever in U5 children. These factors differ in general population compared to and between rural and urban settlements. Although, lack or poor level of education and other factors accounted for delayed care-seeking for febrile U5 children among rural mothers [1], but they have access to recommended treatment of testing fever for malaria parasite before anti-malarial medication. However, the urban women are knowledgeable of malaria, with low prevalence of U5 children with fever, they seek care early but received un-recommended care for malaria fever treatment. These factors and rural–urban disparities shows that ‘one-size-fits-all’ approach to malaria elimination in Igabi LGA may not be effective as when a context-specific malaria intervention is implemented in various communities to drive impact for malaria elimination [4]. For Igabi LGA to achieve greater impact in malaria elimination programme, different interventions for behavioural change should be implemented in rural areas and sub-populations with high burden of U5 fever episode will reduce the number of children dying of malaria [4].

The demographic characteristics in this study were similar to the 2018 Nigeria Demographic Health Survey (DHS) [3]. Further strength of this study lies in the large sample size, being a community survey with sample size well spread in urban and rural settlements of Igabi LGA. The study may not be without limitations. The outcome may not be generalizable to other LGAs in Kaduna State, and it is prone to social desirability and information bias. The information on the last child or youngest child of the mothers was collected to minimize information bias and participants were encouraged to speak the truth, assured of their confidentiality and that no consequences prevailed if they declined to participate. Also, the possibility of error due to mothers’ recall bias on U5 febrile episode in previous two weeks and care-seeking behaviour was minimized with the aid of a pictorial chart and verify hospital cards, prescriptions, drugs receipt, and empty container of the drugs used during the febrile episode. Moreover, the findings are useful for the LGA wherein this study was carried out. Despite these limitations, this study is valuable to identify areas with high burden of fever episode for intervention that will yield high impact for malaria elimination in the LGA.

## Conclusions

Generally, rural–urban disparities existed between fever prevalence in U5 children, care-seeking practices by their mothers, and factors associated with delayed care-seeking and receiving a recommended care for fever. These factors were influenced by differences in rural–urban mothers’ characteristics factors. To rapidly scale up fever treatment for high impact on malaria elimination, the LGA and sub-national Malaria Elimination Programme should employ context-specific interventions rather than ‘one-size-fits-all’ approach for malaria elimination strategy in Kaduna State, Nigeria.

## Data Availability

The datasets used and/or analysed during the current study are available from the corresponding author on reasonable request.

## Abbreviations

ACT: Artemisinin-based combination therapy
LGA: Local government area
RDT: Rapid diagnostic test
WCBA: Women of child-bearing age
MIS: Malaria indicator survey
WHO: World Health Organization
UNICEF: United Nations Children’s Fund
